# Crystal structure of *catena*-poly[[methanoldioxidouranium(VI)]-μ-2-[5-(2-oxidophen­yl)-1*H*-1,2,4-triazol-3-yl]acetato-κ^2^*O*:*O*′]

**DOI:** 10.1107/S2056989024006637

**Published:** 2024-07-12

**Authors:** Oleksandr V. Vashchenko, Dmytro M. Khomenko, Roman O. Doroshchuk, Alexandru-Constantin Stoica, Olga Yu. Vassilyeva, Rostyslav D. Lampeka

**Affiliations:** a Department of Chemistry, Taras Shevchenko National University of Kyiv, Volodymyrska str. 64/13, 01601 Kyiv, Ukraine; b Enamine Ltd. (www.enamine.net), Winston Churchill str. 78, 02094 Kyiv, Ukraine; c "PetruPoni" Institute of Macromolecular Chemistry, Aleea Gr., Ghica Voda 41A, 700487 Iasi, Romania; Illinois State University, USA

**Keywords:** crystal structure, uranyl ion, 1,2,4-triazole, acetate group, hydrogen bonding, LMCT transition

## Abstract

In the title complex, [U(C_10_H_7_N_3_O_3_)O_2_(CH_3_OH)]_*n*_, the U^VI^ cation has a typical penta­gonal–bipyramidal environment with the equatorial plane defined by one N and two O atoms of one doubly deprotonated 2-[5-(2-hy­droxy­phen­yl)-1*H*-1,2,4-triazol-3-yl]acetic acid ligand, a carboxyl­ate O atom of the symmetry-related ligand and the O atom of the methanol mol­ecule [U—N/O_eq_ 2.256 (4)–2.504 (5) Å]. The axial positions are occupied by two oxide O atoms. The equatorial atoms are almost coplanar, with the largest deviation from the mean plane being 0.121 Å for one of the O atoms. The benzene and triazole rings of the tetra­dentate chelating–bridging ligand are twisted by approximately 21.6 (2)° with respect to each other. The carboxyl­ate group of the ligand bridges two uranyl cations, forming a neutral zigzag chain reinforced by a strong O—H⋯O hydrogen bond. In the crystal, adjacent chains are linked into two-dimensional sheets parallel to the *ac* plane by C/N—H⋯N/O hydrogen bonding and π–π inter­actions. Further weak C—H⋯O contacts consolidate the three-dimensional supra­molecular architecture. In the solid state, the compound shows a broad medium intensity LMCT transition centred around 463 nm, which is responsible for its red colour.

## Abstract 2

In [UO_2_*L*(CH_3_OH)]_*n*_, the acetate group of the 1,2,4-triazol-based ligand bridges two uranyl cations, forming a neutral zigzag chain. A solid-state LMCT transition at 463 nm is responsible for the light-red colour of the compound.

## 1. Chemical context

Uranium is the main component of the fuel used in nuclear power reactors for the electricity production. Knowledge of its chemical properties, behaviour, and inter­actions is crucial for the safe and efficient mining extraction process, waste disposal and recycling procedures (Alwaeli & Mannheim, 2022 ▸). Uranium can exist in multiple oxidation states from +3 to +6, depending on its chemical environment and conditions, with the tetra­valent metal being the predominant form in the natural state in many uranium-bearing minerals and ores. In nuclear fuel cycles and certain industrial processes, uranium can also be found in the +6 oxidation state as uranyl ion UO_2_^2+^ or various uranium(VI) compounds. For the research field in chemistry related to the uranium waste management, it remains an important goal to develop hydro­phobic polydentate ligand systems capable of selectively binding actinide ions and transferring them into the organic phase or depositing them on the surface (Ye *et al.*, 2021 ▸; Thuéry & Harrowfield, 2024 ▸).

As our contribution to the field, we have developed convenient synthetic methods to substituted 1,2,4-triazole ligands as potential chelators for uranyl ions (Vashchenko *et al.*, 2020 ▸). The synthesized organic substances have also proved to be useful as analytical reagents for fluorescence determination of UO_2_^2+^ (Vashchenko *et al.*, 2016*a* ▸). 1,2,4-Triazoles bearing free carboxyl­ate ends were considered promising owing to their simultaneous activities as both chelating and bridging ligands that can adopt various coord­ination modes (Vashchenko *et al.*, 2017 ▸). In this study, the crystal structure of [UO_2_*L*(CH_3_OH)]_*n*_, (I), where H_2_*L* is 5-(2-hy­droxy­phen­yl)-1*H*-1,2,4-triazol-3-yl acetic acid, is reported. The title compound was previously published as UO_2_*L*(CH_3_OH)_2_ and studied with IR and NMR spectroscopy but not structurally characterized (Khomenko *et al.*, 2014 ▸). The present structure determination clarified its composition and polymeric arrangement.
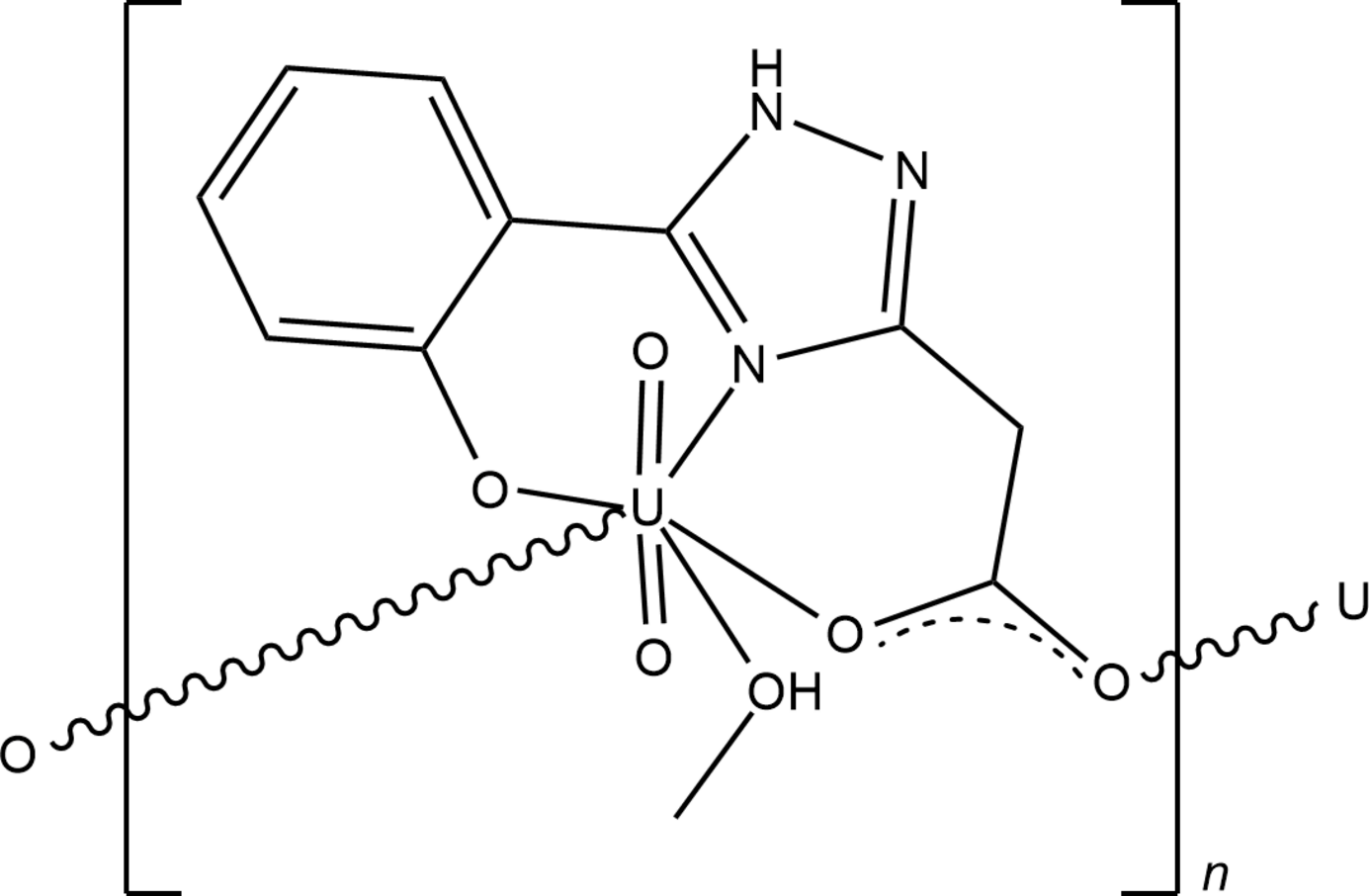


## 2. Structural commentary

**Figure 1:**
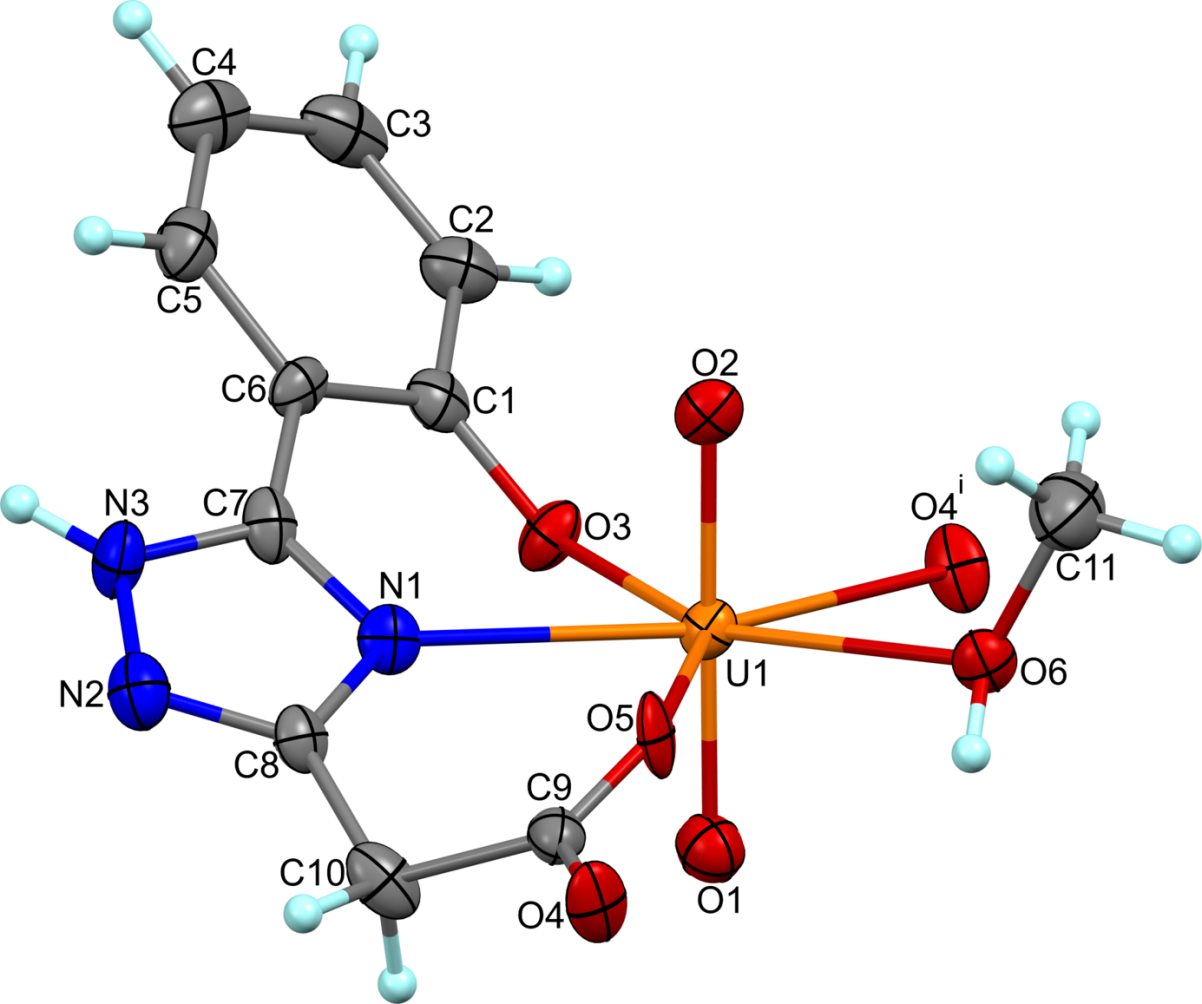
The asymmetric unit of (I) with the atom labelling and displacement ellipsoids at the 50% probability level. The symmetry-equivalent O4 atom is included to complete the coordination sphere of the U^VI^ cation. [Symmetry code: (i) *x* − 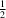, −*y* + 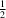, −*z* + 1.]

The repeat motif of (I) consists of a uranyl unit [O=U=O]^2+^, an *L*^2–^ ligand with both carb­oxy­lic acid and phenol groups deprotonated, and a methanol mol­ecule (Fig. 1 ▸). The U^VI^ cation is in a typical penta­gonal–bipyramidal coordination. The uranyl atoms O1 and O2 found at an average distance of 1.761 (5) A from the metal centre form an almost linear O=U=O angle [179.0 (2)°]. The uranyl ion coordinates four O and one N atoms from the two ligands and methanol mol­ecule that occupy the equatorial vertices of the bipyramid with U–N/O_eq_ bond lengths in the range 2.256 (4)–2.504 (5) Å and angles at the metal atom varying from 68.00 (15) to 154.47 (16) (Table 1 ▸). The geometry of the U^VI^ polyhedron is comparable to that in related structures (Raspertova *et al.*, 2012 ▸; Vashchenko *et al.*, 2016*b* ▸). The equatorial atoms are almost coplanar with the largest deviation from the mean plane being 0.121 Å (O6). The benzene and triazole rings of the tetra­dentate ligand are twisted by approximately 21.6 (2)° with respect to each other.

**Figure 2:**
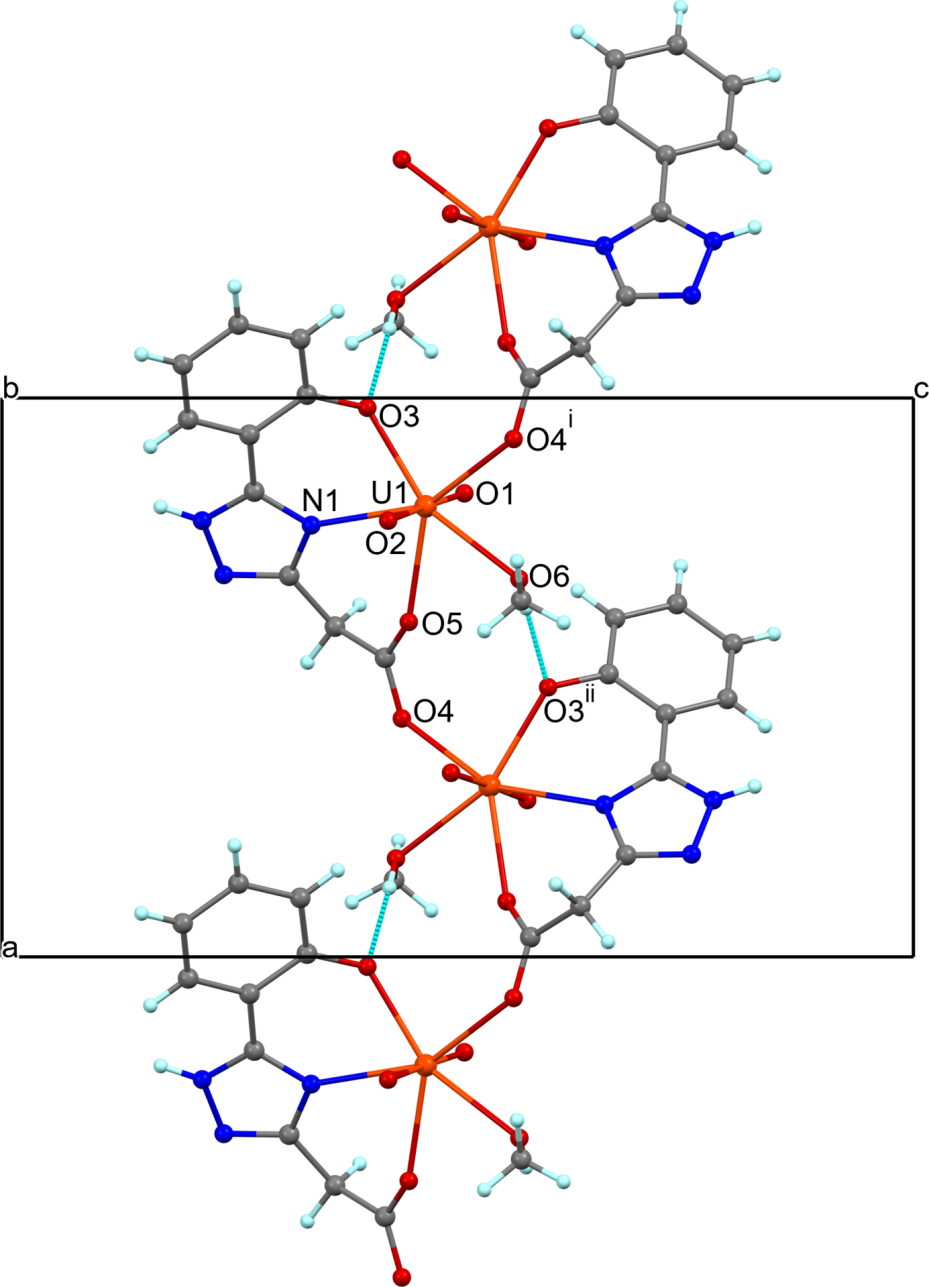
Fragment of the polymeric chain in (I) formed through the ligand carboxylate group bridging of the {UNO_6_} penta­gonal bipyramids and O6—H6⋯O3^ii^ hydrogen bond (blue dashed lines). [Symmetry codes: (i) *x* − 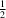, −*y* + 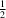, −*z* + 1; (ii) *x* + 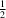, −*y* + 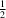, −*z* + 1.]

The C—O bond distances for the carboxyl­ate group [1.253 (7), 1.260 (8) Å] confirm its anionic form. The acetate O4 and O5 atoms act as the bidentate bridging end of the ligand, linking adjacent penta­gonal bipyramids into a neutral zigzag chain running along the *a-*axis direction (Fig. 2 ▸). No sharing of equatorial edges or vertices occurs. A strong inter­molecular hydrogen bond, O6—H6⋯O3^ii^, reinforces the 1D zigzag conformation, generating an 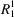
(8) graph-set motif (Bernstein *et al.*, 1995 ▸) (Fig. 2 ▸, Table 2 ▸; symmetry code as given in Table 2 ▸). The closest U⋯U separation within the chain is about 5.69 Å.

## 3. Supra­molecular features

**Figure 3:**
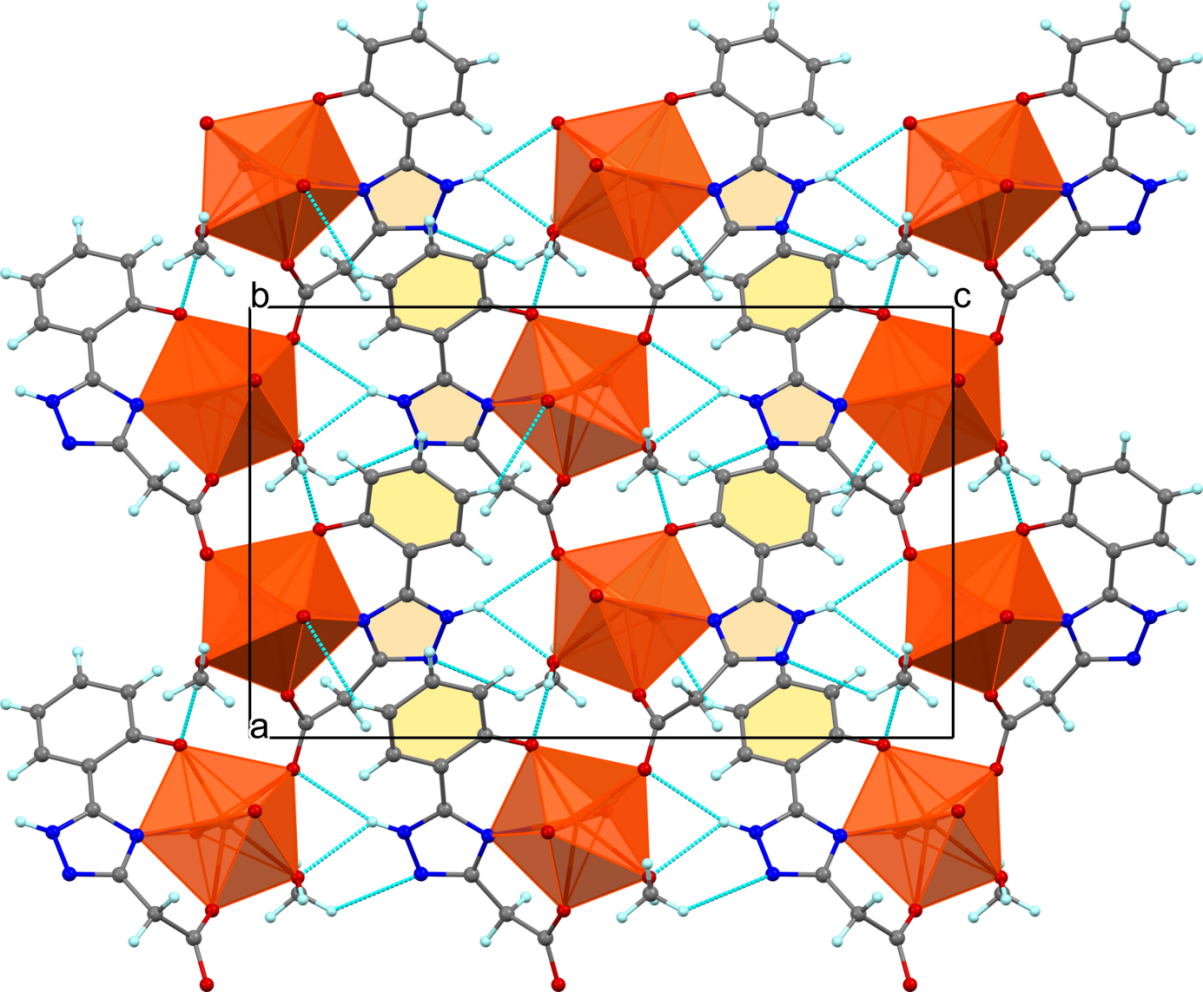
A single plane of (I), viewed along the *b* axis and showing zigzag chains inter­linked by hydrogen bonds and π–π stacking. Orange polyhedra denote U atoms, red spheres O atoms, dark blue spheres N atoms, light blue spheres H atoms; C atoms are grey.

**Figure 4:**
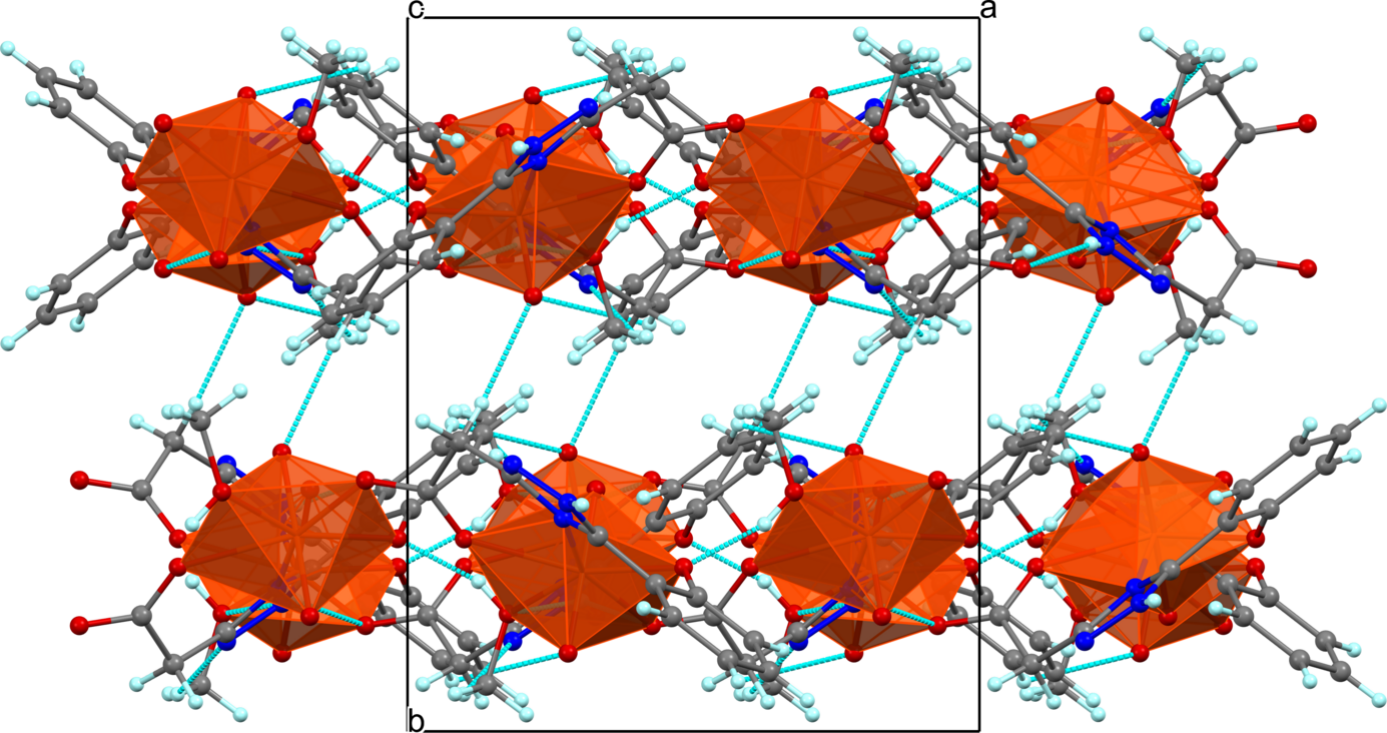
Fragment of the crystal packing of (I) viewed down the *c* axis and showing the sheet-like structure supported by C10—H10*A*⋯O2^v^ hydrogen bond. [Symmetry code: (v) −*x* + 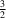, *y* − 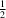, *z*.]

In the solid state, adjacent chains are linked into two-dimensional sheets parallel to the *ac* plane by hydrogen bonding and π–π inter­actions (Fig. 3 ▸). The bifurcated N3—H⋯O and C—H⋯N2 hydrogen bonds involve nitro­gen atoms of the 1,2,4-triazole ring as both the donor and acceptor of protons (Table 2 ▸). The face-to-face aromatic stacking between 1,2,4-triazole and benzene rings from neighbouring chains segments is rather strong, as evidenced by a centroid-to-centroid distance of 3.539 (4) Å, with the tilt angle and ring slippage being 7.1 (4)° and 0.5 Å, respectively. The metal atoms within the sheet are not coplanar, deviating from the mean plane by approximately 0.316 Å on both sides. The sheets inter­act through week C—H⋯O contacts, forming a 3D supra­molecular architecture with distances between the consecutive mean planes corresponding to half the value of the unit-cell parameter *b* (Fig. 4 ▸).

## 4. Database survey

The crystal structures of neither the ligand itself nor its metal complexes are found in the Cambridge Structure Database (CSD, Version 5.45, update of Mar 2024; Groom *et al.*, 2016 ▸). A search of the CSD for structures containing a uranyl ion and the 1,2,4-triazole moiety resulted in twelve hits. Three of them represent metal–organic frameworks (MOFs) with an unsubstituted 1,2,4-triazole ligand and demonstrate remarkable structural features. Ortho­rhom­bic (U^VI^O_2_)_2_[U^VI^O_4_(trz)_2_](OH)_2_, where trz = 1,2,4-triazole (QEKDAN; Weng *et al.*, 2012 ▸), is regarded as containing both a typical uranyl cation and a U^VI^ atom with a coordination polyhedron inter­mediate between a tetra­oxido core and UO_2_^2+^ ion. The neutral 1,2,4-triazole is coordinated to the U^VI^ atom through its N4 atom. The isomorphous ULONOB (Smetana *et al.*, 2021 ▸), which differs from QEKDAN by one hydrogen atom only, was formulated as the mixed-valent uranium complex U^V^O(U^VI^O_2_)_2_(OH)_5_(trz–H)_2_ with the deprotonated 1,2,4-triazole ligand being bound to the U^V^ centre. In ortho­rhom­bic [Hmim][(UO_2_)_2_(trz–H)_5_]·3mim (mim = 1-methyl­imidazole; NULBAA; Smetana *et al.*, 2020 ▸), the uranyl UO_2_^2+^ cations are bridged by five [trz–H]^−^ anions to five other uranyl ions, forming a nearly planar polymeric anionic layer.

While the number of crystal structures of uranyl acetate complexes in the CSD amounts to 125 hits, those with ligands incorporating an acetate functionality in the 1,2,4-triazole ring are limited to three examples. These are Ag^+^/UO_2_^2+^ MOFs based on 1,2,4-triazol-4-yl-acetic acid derivatives (FUHGAT; Senchyk *et al.*, 2020 ▸; SIRYAX and SIRYEB; Senchyk *et al.*, 2022 ▸). The compounds have uranium(VI) in a penta­gonal–bipyramidal {UO_7_} arrangement similar to (I), and are distinguished by the acetato group coordination mode, which provides exclusively monodentate coordination to uranyl ions. Further examples of uranyl complexes with the ligands combining 1,2,4-triazole moiety and carboxyl­ate groups include pure uranyl, and heterometallic Zn^2+^/UO_2_^2+^ and Cd^2+^/UO_2_^2+^ coordination polymers based on the 4-(4′-carb­oxy­phen­yl)-1,2,4-triazole ligand (XIKFOP, XIKFEF and XIKFIJ, respectively; Zhao *et al.*, 2018 ▸).

Three last hits of the twelve structures are mol­ecular uranyl complexes where, depending on the organic substituents positions in the 1,2,4-triazole moiety, triazole-N1 (MIDXEC; Daro *et al.*, 2001 ▸) or N4 coordination (WAWROD; Raspertova *et al.*, 2012 ▸; XUYKOT; Vashchenko *et al.*, 2016*b* ▸) is realized.

## 5. Synthesis and crystallization

**Figure 5:**
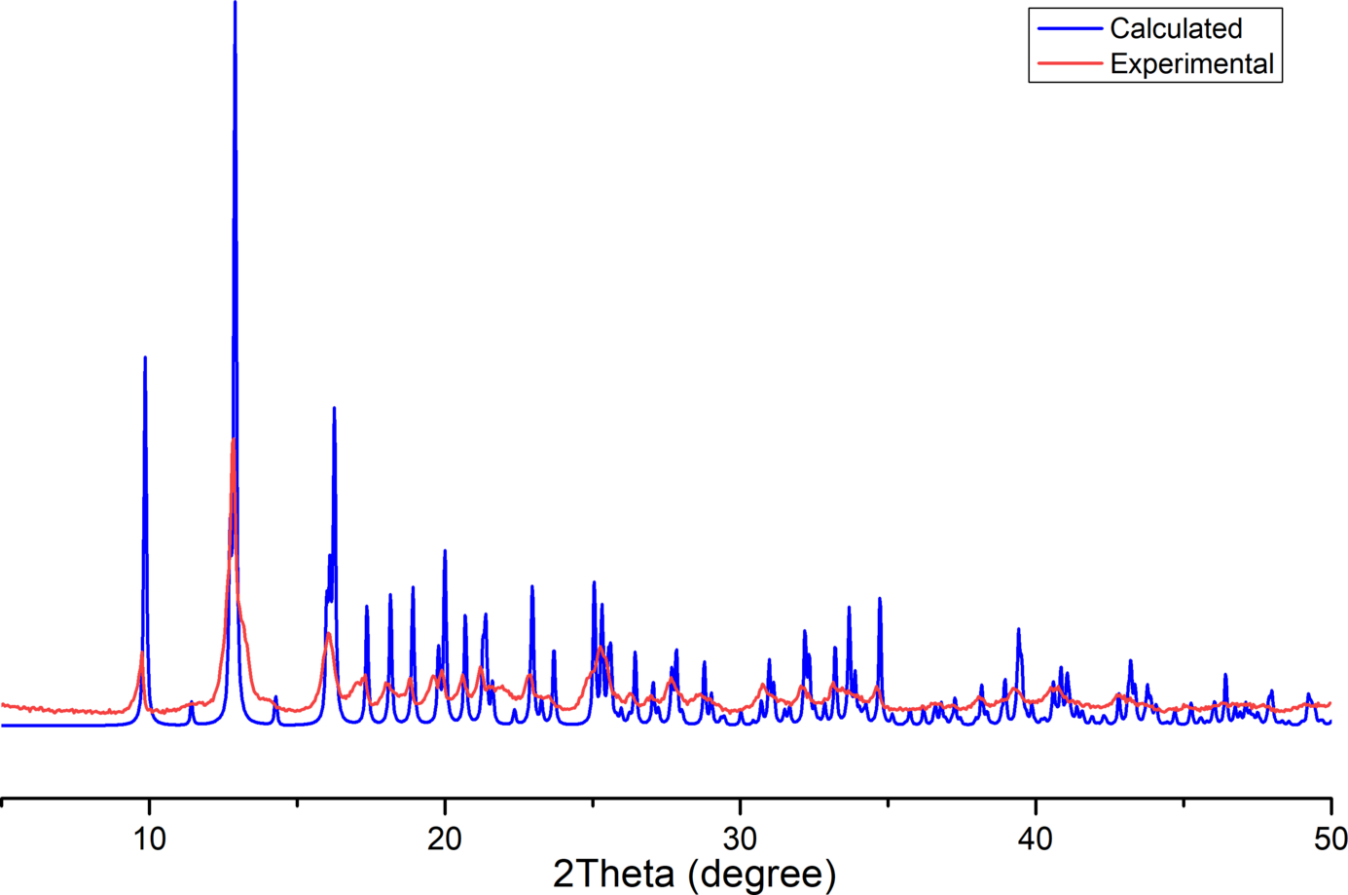
Powder XRD patterns of (I).

The title compound was synthesized according to the previously published method (Khomenko *et al.*, 2014 ▸). X-ray quality light-red crystals were obtained by slow crystallization from the reaction mixture. Phase purity was confirmed by comparing the observed and calculated powder X-ray diffraction patterns (Fig. 5 ▸). The PXRD pattern was acquired on a Shimadzu XRD-6000 diffractometer using Cu *K*α radiation (5–50° range, 0.05° step). The main features of the IR and ^1^H NMR spectra of (I) were in satisfactory agreement with those reported before.

**Figure 6:**
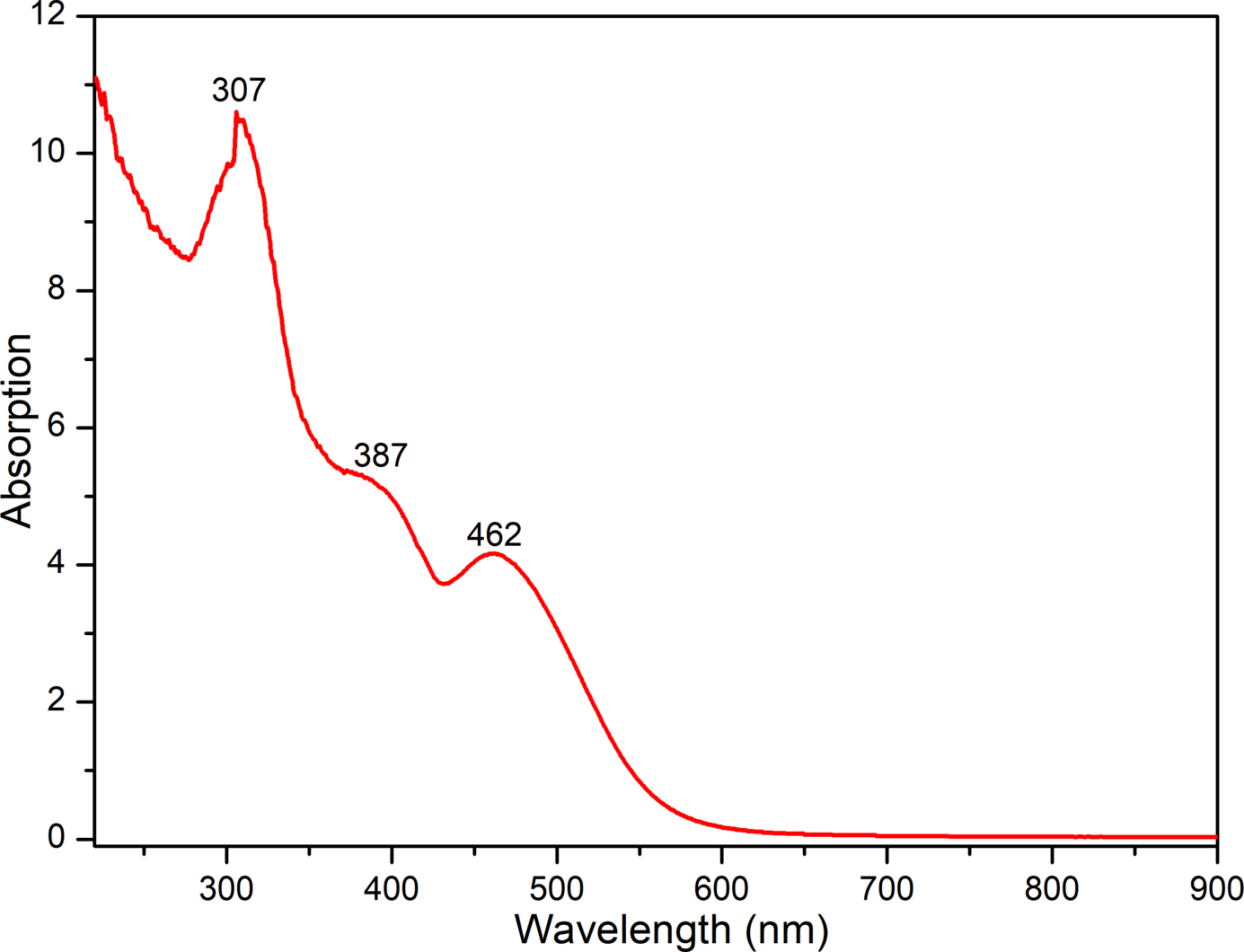
The solid-state UV-Vis absorption spectrum of (I) at room temperature.

The UV-Vis absorption spectrum was measured in a diffuse reflectance mode on a Shimadzu UV-2600i spectrophotometer using a powdered microcrystalline sample of (I) at ambient temperature (Fig. 6 ▸). The broad unstructured absorption band of medium intensity in the visible region observed at 463 nm can be assigned to O_2p_ → U_5f_ LMCT transitions between the filled O-atom orbitals of the coordinated *L*^2–^ ligand and the empty orbitals of the U^VI^ ion (Azam *et al.*, 2016 ▸). The band gradually slopes into the green–blue region of the spectrum, being responsible for the red colour of (I). The shoulder visible around 387 nm is likely due to the charge transfer within U=O double bonds (Sladkov *et al.*, 2018 ▸). The strong and narrow band at 307 nm is attributed to the π → π* transition within the aromatic ligand. The electronic structure of (I) is significantly different from that of typical uranyl compounds that show an intense LMCT transition with a well-deﬁned vibrational fine structure centred around 420 nm (Natrajan, 2012 ▸).

## 6. Refinement

Crystal data, data collection and structure refinement details are summarized in Table 3 ▸. Anisotropic displacement parameters were employed for the non-hydrogen atoms. The residual electron density in the vicinities of atoms O4, O5 and C10 suggested some disorder in this part of the ligand, but a suitable model for refining the disorder was not found. The H atom bound to O was found in a difference-Fourier map and refined with the bond distance fixed at 0.85 (1) Å and *U*_iso_(H) = 1.5*U*_eq_O. The remaining H atoms were placed in calculated positions and refined using a riding model with isotropic displacement parameters based on those of the parent atom [C—H = 0.95/0.99 Å, N—H = 0.88 Å, *U*_iso_(H) = 1.2*U*_eq_C/N for CH, CH_2_ and NH, respectively; C—H = 0.98 Å, *U*_iso_(H) = 1.5*U*_eq_C for CH_3_]. The idealized methyl group was refined as a rotating group.

## Supplementary Material

Crystal structure: contains datablock(s) I. DOI: 10.1107/S2056989024006637/ej2005sup1.cif

Structure factors: contains datablock(s) I. DOI: 10.1107/S2056989024006637/ej2005Isup2.hkl

CCDC reference: 2368205

Additional supporting information:  crystallographic information; 3D view; checkCIF report

## Figures and Tables

**Table 1:** Selected geometric parameters (Å, °)

U1—O1	1.764 (5)	U1—O5	2.382 (5)
U1—O2	1.757 (5)	U1—O6	2.474 (5)
U1—O3	2.256 (4)	U1—N1	2.504 (5)
U1—O4^i^	2.415 (4)		
			
O1—U1—O3	93.63 (19)	O3—U1—O4^i^	84.70 (15)
O1—U1—O4^i^	89.78 (19)	O3—U1—O5	137.78 (15)
O1—U1—O5	88.01 (19)	O3—U1—O6	152.73 (16)
O1—U1—O6	93.32 (19)	O3—U1—N1	69.78 (16)
O1—U1—N1	92.6 (2)	O4^i^—U1—O6	68.98 (15)
O2—U1—O1	179.0 (2)	O4^i^—U1—N1	154.47 (16)
O2—U1—O3	87.38 (18)	O5—U1—O4^i^	137.52 (15)
O2—U1—O4^i^	90.19 (18)	O5—U1—O6	68.81 (14)
O2—U1—O5	91.33 (18)	O5—U1—N1	68.00 (15)
O2—U1—O6	85.72 (19)	O6—U1—N1	136.12 (15)
O2—U1—N1	87.88 (19)		

Symmetry code: (i) 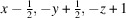.

**Table 2:** Hydrogen-bond geometry (Å, °)

*D*—H⋯*A*	*D*—H	H⋯*A*	*D*⋯*A*	*D*—H⋯*A*
O6—H6⋯O3^ii^	0.85 (1)	1.82 (3)	2.634 (6)	160 (7)
N3—H3⋯O4^iii^	0.88	2.45	3.291 (7)	159
N3—H3⋯O6^iv^	0.88	2.38	2.966 (7)	124
C10—H10*A*⋯O2^v^	0.99	2.34	3.300 (8)	163
C11—H11*A*⋯N2^vi^	0.98	2.66	3.266 (10)	120

Symmetry codes: (ii) 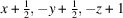; (iii) 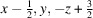; (iv) 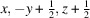; (v) 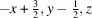; (vi) 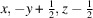.

**Table 3:** Experimental details

Crystal data	
Chemical formula	[U(C_10_H_7_N_3_O_3_)O_2_(CH_4_O)]
*M*_r_	519.26
Crystal system, space group	Orthorhombic, *Pbca*
Temperature (K)	200
*a*, *b*, *c* (Å)	10.9966 (6), 13.7147 (10), 17.9345 (9)
*V* (Å^3^)	2704.8 (3)
*Z*	8
Radiation type	Mo *K*α
μ (mm^−1^)	12.03
Crystal size (mm)	0.15 × 0.1 × 0.1

Data collection	
Diffractometer	Xcalibur, Eos
Absorption correction	Multi-scan (*CrysAlis PRO*; Rigaku OD, 2023 ▸)
*T*_min_, *T*_max_	0.503, 1.000
No. of measured, independent and observed [*I* > 2σ(*I*)] reflections	6980, 2380, 1904
*R*_int_	0.040
(sin θ/λ)_max_ (Å^−1^)	0.595

Refinement	
*R*[*F*^2^ > 2σ(*F*^2^)], *wR*(*F*^2^), *S*	0.033, 0.056, 1.05
No. of reflections	2380
No. of parameters	194
No. of restraints	1
H-atom treatment	H atoms treated by a mixture of independent and constrained refinement
Δρ_max_, Δρ_min_ (e Å^−3^)	1.08, −0.82

Computer programs: *CrysAlis PRO* (Rigaku OD, 2023 ▸), *SHELXT2014/5* (Sheldrick, 2015*a* ▸), *SHELXL2018/3* (Sheldrick, 2015*b* ▸), *Mercury* (Macrae *et al.*, 2020 ▸) and *OLEX2* (Dolomanov *et al.*, 2009 ▸).

## supplementary crystallographic information

### catena-Poly[[methanoldioxidouranium(VI)]-µ-2-[5-(2-oxidophenyl)-1H-1,2,4-triazol-3-yl]acetato-κ2O:O'] Crystal data

**Table:**

[U(C_10_H_7_N_3_O_3_)O_2_(CH_4_O)]	*D*_x_ = 2.550 Mg m^−^^3^
*M**_r_* = 519.26	Mo *K*α radiation, λ = 0.71073 Å
Orthorhombic, *P**b**c**a*	Cell parameters from 2366 reflections
*a* = 10.9966 (6) Å	θ = 2.6–30.7°
*b* = 13.7147 (10) Å	µ = 12.03 mm^−^^1^
*c* = 17.9345 (9) Å	*T* = 200 K
*V* = 2704.8 (3) Å^3^	Prism, clear light red
*Z* = 8	0.15 × 0.1 × 0.1 mm
*F*(000) = 1904	

### catena-Poly[[methanoldioxidouranium(VI)]-µ-2-[5-(2-oxidophenyl)-1H-1,2,4-triazol-3-yl]acetato-κ2O:O'] Data collection

**Table:**

Xcalibur, Eos diffractometer	2380 independent reflections
Radiation source: fine-focus sealed X-ray tube, Enhance (Mo) X-ray Source	1904 reflections with *I* > 2σ(*I*)
Graphite monochromator	*R*_int_ = 0.040
Detector resolution: 8.0797 pixels mm^-1^	θ_max_ = 25.0°, θ_min_ = 2.3°
ω scans	*h* = −13→7
Absorption correction: multi-scan (CrysAlisPro; Rigaku OD, 2023)	*k* = −16→14
*T*_min_ = 0.503, *T*_max_ = 1.000	*l* = −11→21
6980 measured reflections	

### catena-Poly[[methanoldioxidouranium(VI)]-µ-2-[5-(2-oxidophenyl)-1H-1,2,4-triazol-3-yl]acetato-κ2O:O'] Refinement

**Table:**

Refinement on *F*^2^	Primary atom site location: dual
Least-squares matrix: full	Hydrogen site location: mixed
*R*[*F*^2^ > 2σ(*F*^2^)] = 0.033	H atoms treated by a mixture of independent and constrained refinement
*wR*(*F*^2^) = 0.056	*w* = 1/[σ^2^(*F*_o_^2^) + (0.0147*P*)^2^] where *P* = (*F*_o_^2^ + 2*F*_c_^2^)/3
*S* = 1.05	(Δ/σ)_max_ = 0.002
2380 reflections	Δρ_max_ = 1.08 e Å^−^^3^
194 parameters	Δρ_min_ = −0.82 e Å^−^^3^
1 restraint	

### catena-Poly[[methanoldioxidouranium(VI)]-µ-2-[5-(2-oxidophenyl)-1H-1,2,4-triazol-3-yl]acetato-κ2O:O'] Special details

**Table:**

Geometry. All esds (except the esd in the dihedral angle between two l.s. planes) are estimated using the full covariance matrix. The cell esds are taken into account individually in the estimation of esds in distances, angles and torsion angles; correlations between esds in cell parameters are only used when they are defined by crystal symmetry. An approximate (isotropic) treatment of cell esds is used for estimating esds involving l.s. planes.

### catena-Poly[[methanoldioxidouranium(VI)]-µ-2-[5-(2-oxidophenyl)-1H-1,2,4-triazol-3-yl]acetato-κ2O:O'] Fractional atomic coordinates and isotropic or equivalent isotropic displacement parameters (Å^2^)

**Table:**

	*x*	*y*	*z*	*U*_iso_*/*U*_eq_	
U1	0.69391 (2)	0.27580 (2)	0.53512 (2)	0.01841 (9)	
O1	0.6706 (4)	0.1612 (4)	0.4927 (2)	0.0292 (13)	
O2	0.7194 (4)	0.3902 (4)	0.5763 (2)	0.0237 (12)	
O3	0.5175 (4)	0.2700 (4)	0.5992 (2)	0.0249 (12)	
O4	1.0734 (4)	0.1482 (4)	0.5613 (2)	0.0274 (13)	
O5	0.9007 (4)	0.2302 (4)	0.5537 (2)	0.0258 (12)	
O6	0.8241 (4)	0.3355 (4)	0.4323 (3)	0.0235 (12)	
H6	0.875 (5)	0.290 (4)	0.425 (4)	0.035*	
N1	0.7276 (5)	0.2011 (4)	0.6608 (3)	0.0212 (15)	
N2	0.8163 (5)	0.1263 (5)	0.7566 (3)	0.0300 (15)	
N3	0.7195 (5)	0.1797 (5)	0.7804 (3)	0.0278 (15)	
H3	0.694586	0.183100	0.826934	0.033*	
C1	0.4940 (6)	0.3117 (5)	0.6654 (4)	0.0226 (17)	
C2	0.3944 (6)	0.3736 (5)	0.6727 (4)	0.0285 (19)	
H2	0.343935	0.386412	0.630816	0.034*	
C3	0.3686 (7)	0.4162 (6)	0.7405 (5)	0.036 (2)	
H3A	0.299727	0.457636	0.744780	0.043*	
C4	0.4401 (6)	0.4000 (6)	0.8018 (4)	0.035 (2)	
H4	0.421840	0.430771	0.847916	0.043*	
C5	0.5383 (7)	0.3390 (6)	0.7957 (4)	0.0295 (19)	
H5	0.588149	0.327390	0.838037	0.035*	
C6	0.5658 (6)	0.2939 (5)	0.7285 (3)	0.0211 (17)	
C7	0.6674 (6)	0.2263 (5)	0.7233 (4)	0.0233 (17)	
C8	0.8180 (6)	0.1388 (5)	0.6849 (4)	0.0205 (16)	
C9	0.9648 (6)	0.1620 (5)	0.5797 (4)	0.0211 (17)	
C10	0.9090 (6)	0.0939 (5)	0.6347 (4)	0.0284 (19)	
H10A	0.869498	0.039972	0.607091	0.034*	
H10B	0.974574	0.065223	0.665466	0.034*	
C11	0.8620 (7)	0.4352 (6)	0.4303 (4)	0.042 (2)	
H11A	0.902753	0.448521	0.382807	0.063*	
H11B	0.918432	0.447745	0.471518	0.063*	
H11C	0.790796	0.477681	0.435274	0.063*	

### catena-Poly[[methanoldioxidouranium(VI)]-µ-2-[5-(2-oxidophenyl)-1H-1,2,4-triazol-3-yl]acetato-κ2O:O'] Atomic displacement parameters (Å^2^)

**Table:**

	*U*^11^	*U*^22^	*U*^33^	*U*^12^	*U*^13^	*U*^23^
U1	0.01371 (14)	0.02400 (17)	0.01753 (13)	0.00033 (12)	−0.00102 (12)	−0.00098 (14)
O1	0.030 (3)	0.028 (3)	0.030 (3)	0.000 (2)	−0.003 (2)	0.002 (3)
O2	0.015 (3)	0.030 (3)	0.025 (3)	0.003 (2)	−0.001 (2)	−0.002 (2)
O3	0.019 (2)	0.038 (3)	0.018 (2)	0.001 (2)	−0.002 (2)	−0.004 (3)
O4	0.014 (3)	0.038 (4)	0.031 (3)	0.007 (2)	0.011 (2)	0.011 (3)
O5	0.020 (3)	0.035 (3)	0.022 (2)	−0.003 (2)	0.006 (2)	0.018 (3)
O6	0.018 (3)	0.026 (3)	0.027 (2)	0.003 (2)	0.003 (2)	0.002 (3)
N1	0.011 (3)	0.027 (4)	0.026 (3)	−0.006 (2)	0.002 (3)	0.002 (3)
N2	0.029 (4)	0.032 (4)	0.028 (3)	−0.004 (3)	−0.001 (3)	0.006 (3)
N3	0.027 (4)	0.039 (4)	0.017 (3)	0.003 (3)	0.001 (3)	0.006 (3)
C1	0.018 (4)	0.020 (5)	0.029 (4)	−0.006 (3)	0.004 (3)	0.003 (4)
C2	0.022 (4)	0.025 (5)	0.039 (4)	−0.002 (3)	0.002 (4)	−0.001 (4)
C3	0.024 (4)	0.025 (5)	0.059 (6)	−0.002 (4)	0.016 (4)	0.004 (5)
C4	0.030 (5)	0.034 (5)	0.042 (5)	−0.013 (4)	0.019 (4)	−0.005 (5)
C5	0.032 (5)	0.033 (5)	0.024 (4)	−0.012 (4)	0.002 (4)	0.000 (4)
C6	0.019 (4)	0.024 (5)	0.020 (3)	−0.006 (3)	0.007 (3)	−0.003 (3)
C7	0.018 (4)	0.029 (5)	0.023 (4)	−0.008 (3)	−0.002 (3)	0.007 (4)
C8	0.016 (4)	0.023 (4)	0.023 (4)	−0.006 (3)	0.002 (3)	0.005 (4)
C9	0.024 (4)	0.019 (5)	0.020 (4)	−0.004 (3)	−0.001 (3)	0.000 (4)
C10	0.015 (4)	0.025 (5)	0.044 (5)	−0.004 (3)	0.000 (4)	0.009 (4)
C11	0.051 (5)	0.038 (6)	0.037 (5)	−0.012 (5)	0.006 (4)	−0.001 (5)

### catena-Poly[[methanoldioxidouranium(VI)]-µ-2-[5-(2-oxidophenyl)-1H-1,2,4-triazol-3-yl]acetato-κ2O:O'] Geometric parameters (Å, º)

**Table:**

U1—O1	1.764 (5)	C1—C2	1.392 (9)
U1—O2	1.757 (5)	C1—C6	1.401 (9)
U1—O3	2.256 (4)	C2—H2	0.9500
U1—O4^i^	2.415 (4)	C2—C3	1.379 (10)
U1—O5	2.382 (5)	C3—H3A	0.9500
U1—O6	2.474 (5)	C3—C4	1.369 (10)
U1—N1	2.504 (5)	C4—H4	0.9500
O3—C1	1.343 (8)	C4—C5	1.371 (10)
O4—C9	1.253 (7)	C5—H5	0.9500
O5—C9	1.260 (8)	C5—C6	1.389 (9)
O6—H6	0.849 (10)	C6—C7	1.454 (9)
O6—C11	1.430 (8)	C8—C10	1.481 (9)
N1—C7	1.347 (8)	C9—C10	1.490 (9)
N1—C8	1.380 (8)	C10—H10A	0.9900
N2—N3	1.361 (8)	C10—H10B	0.9900
N2—C8	1.298 (8)	C11—H11A	0.9800
N3—H3	0.8800	C11—H11B	0.9800
N3—C7	1.335 (8)	C11—H11C	0.9800
			
O1—U1—O3	93.63 (19)	C1—C2—H2	119.9
O1—U1—O4^i^	89.78 (19)	C3—C2—C1	120.2 (7)
O1—U1—O5	88.01 (19)	C3—C2—H2	119.9
O1—U1—O6	93.32 (19)	C2—C3—H3A	119.3
O1—U1—N1	92.6 (2)	C4—C3—C2	121.4 (7)
O2—U1—O1	179.0 (2)	C4—C3—H3A	119.3
O2—U1—O3	87.38 (18)	C3—C4—H4	120.4
O2—U1—O4^i^	90.19 (18)	C3—C4—C5	119.2 (8)
O2—U1—O5	91.33 (18)	C5—C4—H4	120.4
O2—U1—O6	85.72 (19)	C4—C5—H5	119.6
O2—U1—N1	87.88 (19)	C4—C5—C6	120.9 (7)
O3—U1—O4^i^	84.70 (15)	C6—C5—H5	119.6
O3—U1—O5	137.78 (15)	C1—C6—C7	119.5 (6)
O3—U1—O6	152.73 (16)	C5—C6—C1	120.0 (6)
O3—U1—N1	69.78 (16)	C5—C6—C7	120.4 (6)
O4^i^—U1—O6	68.98 (15)	N1—C7—C6	126.4 (6)
O4^i^—U1—N1	154.47 (16)	N3—C7—N1	107.7 (6)
O5—U1—O4^i^	137.52 (15)	N3—C7—C6	125.8 (6)
O5—U1—O6	68.81 (14)	N1—C8—C10	123.7 (6)
O5—U1—N1	68.00 (15)	N2—C8—N1	112.4 (6)
O6—U1—N1	136.12 (15)	N2—C8—C10	123.9 (7)
C1—O3—U1	126.9 (4)	O4—C9—O5	123.2 (6)
C9—O4—U1^ii^	130.3 (4)	O4—C9—C10	118.1 (6)
C9—O5—U1	141.3 (4)	O5—C9—C10	118.7 (6)
U1—O6—H6	105 (5)	C8—C10—C9	114.8 (6)
C11—O6—U1	120.3 (4)	C8—C10—H10A	108.6
C11—O6—H6	120 (5)	C8—C10—H10B	108.6
C7—N1—U1	124.9 (4)	C9—C10—H10A	108.6
C7—N1—C8	104.7 (6)	C9—C10—H10B	108.6
C8—N1—U1	129.9 (4)	H10A—C10—H10B	107.5
C8—N2—N3	104.5 (6)	O6—C11—H11A	109.5
N2—N3—H3	124.7	O6—C11—H11B	109.5
C7—N3—N2	110.7 (6)	O6—C11—H11C	109.5
C7—N3—H3	124.7	H11A—C11—H11B	109.5
O3—C1—C2	119.6 (7)	H11A—C11—H11C	109.5
O3—C1—C6	122.1 (6)	H11B—C11—H11C	109.5
C2—C1—C6	118.3 (7)		
			
U1—O3—C1—C2	125.8 (6)	N3—N2—C8—N1	1.8 (8)
U1—O3—C1—C6	−55.2 (9)	N3—N2—C8—C10	180.0 (6)
U1^ii^—O4—C9—O5	−8.3 (10)	C1—C2—C3—C4	0.7 (11)
U1^ii^—O4—C9—C10	171.3 (4)	C1—C6—C7—N1	23.3 (11)
U1—O5—C9—O4	158.0 (5)	C1—C6—C7—N3	−158.3 (7)
U1—O5—C9—C10	−21.7 (11)	C2—C1—C6—C5	−1.3 (10)
U1—N1—C7—N3	−172.7 (4)	C2—C1—C6—C7	177.4 (6)
U1—N1—C7—C6	5.9 (10)	C2—C3—C4—C5	−1.0 (11)
U1—N1—C8—N2	170.6 (4)	C3—C4—C5—C6	0.1 (11)
U1—N1—C8—C10	−7.6 (10)	C4—C5—C6—C1	1.1 (11)
O3—C1—C2—C3	179.4 (6)	C4—C5—C6—C7	−177.6 (7)
O3—C1—C6—C5	179.7 (6)	C5—C6—C7—N1	−158.0 (7)
O3—C1—C6—C7	−1.6 (10)	C5—C6—C7—N3	20.4 (11)
O4—C9—C10—C8	146.7 (6)	C6—C1—C2—C3	0.5 (11)
O5—C9—C10—C8	−33.6 (9)	C7—N1—C8—N2	−0.6 (8)
N1—C8—C10—C9	45.3 (9)	C7—N1—C8—C10	−178.8 (6)
N2—N3—C7—N1	2.0 (8)	C8—N1—C7—N3	−0.9 (8)
N2—N3—C7—C6	−176.6 (6)	C8—N1—C7—C6	177.8 (7)
N2—C8—C10—C9	−132.7 (7)	C8—N2—N3—C7	−2.3 (8)

Symmetry codes: (i) *x*−1/2, −*y*+1/2, −*z*+1; (ii) *x*+1/2, −*y*+1/2, −*z*+1.

### catena-Poly[[methanoldioxidouranium(VI)]-µ-2-[5-(2-oxidophenyl)-1H-1,2,4-triazol-3-yl]acetato-κ2O:O'] Hydrogen-bond geometry (Å, º)

**Table:**

*D*—H···*A*	*D*—H	H···*A*	*D*···*A*	*D*—H···*A*
O6—H6···O3^ii^	0.85 (1)	1.82 (3)	2.634 (6)	160 (7)
N3—H3···O4^iii^	0.88	2.45	3.291 (7)	159
N3—H3···O6^iv^	0.88	2.38	2.966 (7)	124
C10—H10*A*···O2^v^	0.99	2.34	3.300 (8)	163
C11—H11*A*···N2^vi^	0.98	2.66	3.266 (10)	120

Symmetry codes: (ii) *x*+1/2, −*y*+1/2, −*z*+1; (iii) *x*−1/2, *y*, −*z*+3/2; (iv) *x*, −*y*+1/2, *z*+1/2; (v) −*x*+3/2, *y*−1/2, *z*; (vi) *x*, −*y*+1/2, *z*−1/2.
